# Supplementary material for: Z-Scheme Heterojunction of 3-Dimensional Hierarchical Bi3O4Cl/Bi5O7I for a Significant Enhancement in the Photocatalytic Degradation of Organic Pollutants (RhB and BPA)
Source: Nanomaterials (Basel). 2022 Feb 24;12(5):767. doi: 10.3390/nano12050767 (PMC8911787; doi:10.3390/nano12050767)
Supplement: Supplementary file 1 [file nanomaterials-12-00767-s001.zip › nanomaterials-1612865-supplementary.pdf]

nanomaterials

Supplementary material

Z-Scheme Heterojunction of 3-Dimensional Hierarchical Bi_3O_4Cl/Bi_5O_7I for a Significant Enhancement in the Photocatalytic Degradation of Organic Pollutants (RhB and BPA)

Syed Taj Ud Din, Hankyu Lee and Woochul Yang *

Department of Physics, Dongguk University, Seoul 04620, Korea; tajuddins.phy@gmail.com (S.T.U.D.); lhk0022@naver.com (H.L.)
* Correspondence: wyang@dongguk.edu; Tel.: +82-222-603-444

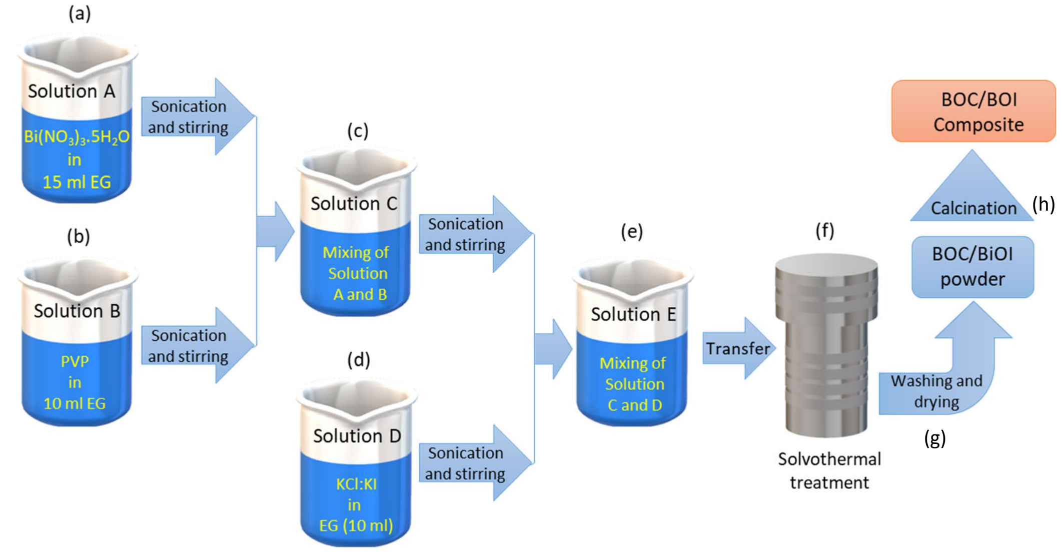

(a). Dissolution Of Bismuth Nitrate in 15 ml EG to prepare Solution A
(b). Dissolution of PVP in 10 ml EG to prepare Solution B
(c). Mixing of sol A and Sol B, to prepare solution C.
(d). Dissolution of KCl and KI with Different ratio in 10 ml EG to prepare solution D
(e). Mixing of solution C and solution D, to prepare solution E
(f). Hydrothermal treatment at 160C for 12 hour
(g). Washing with water and drying over night.
(h). Calcination at 450 C for 1 hour to obtain the target BOC/BOI

Figure S1. Steps involved in the synthesis of heterostructure BOC/BOI composite.

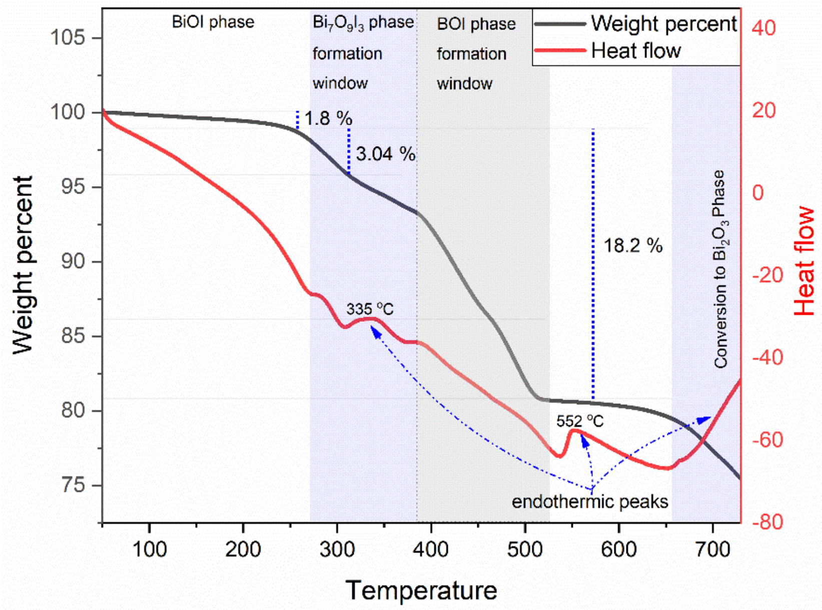

Figure S2. TGA and DSC plot of BiOI photocatalyst.

1.Thermogravimetric analysis

In the calcination process, TGA is an effective technique to observe the phase transformation and weight-loss% in a material. Thus, the specific calcination temperature for the desired phase formation of our targeted materials was determined through the TGA measurement. This measurement was conducted in the temperature range from 50 °C to 750 °C of the hydrothermally prepared BiOI raw powder. Figure S2 shows the TGA (weight-loss%) and DSC (endothermic and exothermic) plot of the raw powder. The obtained TGA plot is divided into four segments, wherein segment I, segment II, segment III, and segment IV correspond to the temperature ranging from 50 °C to 270 °C, from 270 °C to 390 °C, from 390 °C to 530 °C, and from 530 °C to 620 °C, respectively. In segment I, it can be observed that BiOI lost 1.8% of its initial weight due to the existence of water molecules and volatile compounds [1,2]. In segment II, the decomposition of EG molecules and deiodination occurred, which reduced the weight by 3.8% [3,4]. Due to deiodination in this segment, the heat flow curve showed a small endothermic peak at 335 °C, indicating that the BiOI phase is transformed into $Bi_7O_9I_3$ [5]. In Segment III, it can be observed that the deiodination process continued, which in turn reduced the weight by 18.02% and created an endothermic peak at 552 °C, confirming the phase transformation from $Bi_7O_9I_3$ to Bi_5O_7I (BOI) [6]. Lastly, in segment IV, it is observed that there is no loss in weight %, thus showing the thermal stability of the targeted BOI photocatalyst. However, it can be seen that by increasing the temperature above 620 °C, the loss in weight % is re-started. Further, a dominant endothermic peak at 650 °C is created, which confirmed the conversion of BOI to the Bi_2O_3 phase [7]. Thus, based on TGA analysis, it is confirmed that the targeted phase of the heterostructured composite, i.e., the BOC/BOI composite, can be formed when the BOC/BiOI raw powder is calcined for 1 h at 450 °C [8].

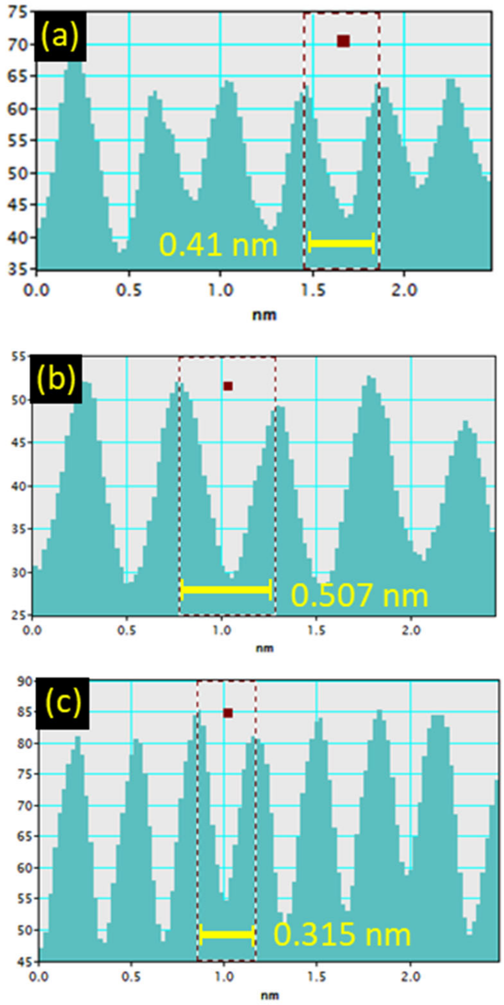

Figure S3. Intensity line profiles of the inverse FFT of (**a**, **b**) BOC and BOI (**c**) in the BOC/BOI heterostructure.

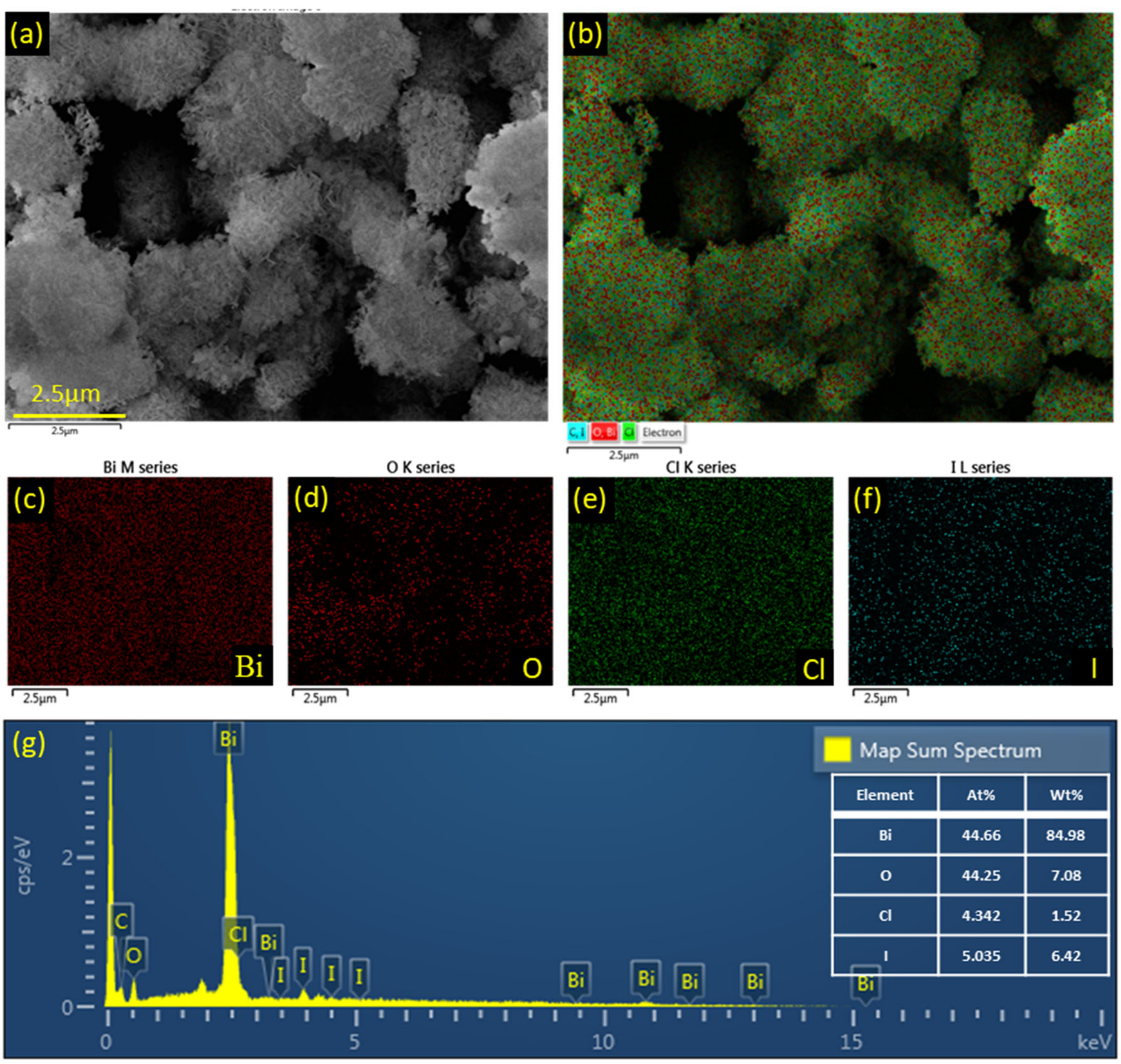

Figure S4. FESEM image of (**a**) BOC/BOI-3 composite and (**b**) its corresponding EDS sum image consists of four different colors: light red, dark red, green, and blue; these denote the presence of (**c**) Bi, (**d**) O, (**e**) Cl, and (**f**) I in the BOC/BOI-3 heterostructure, respectively (**g**) EDS spectrum of BOC/BOI-3 composite, with the inset table showing the atomic and elemental weight percentages of BOC/BOI-3 nano composite.

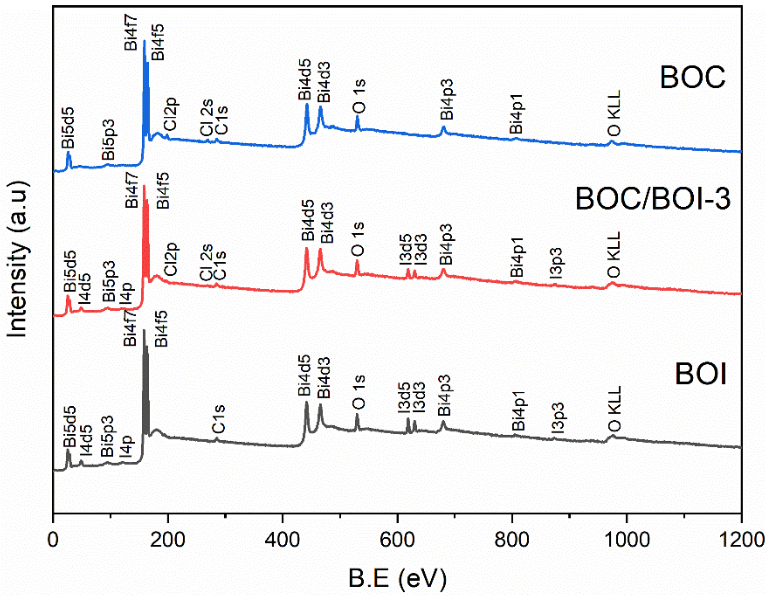

Figure S5. XPS survey spectra for BOC, BOI, and BOC/BOI based photocatalysts.

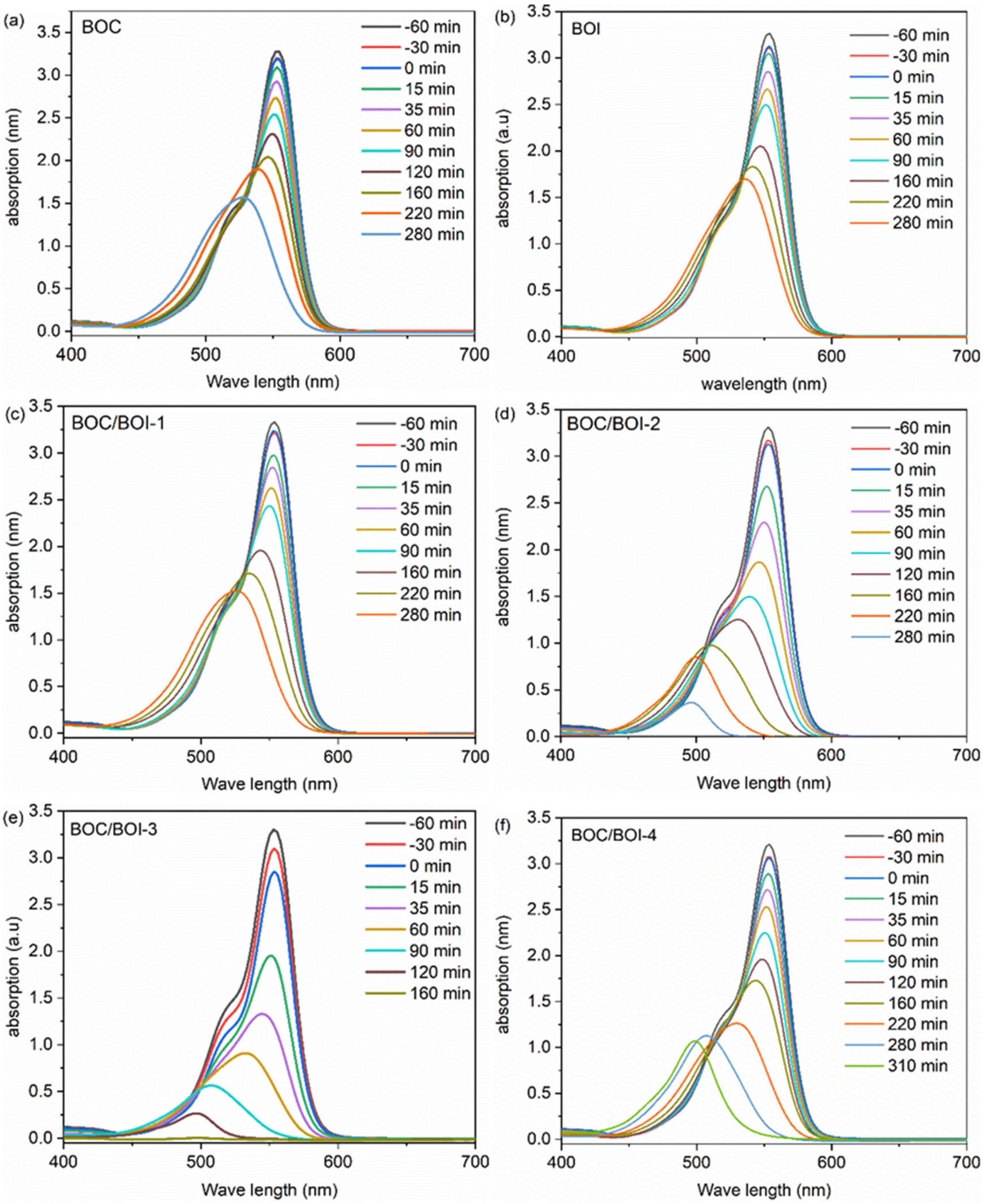

Figure S6. Time resolved absorption spectra of RhB degradation with (**a**) BOC, (**b**) BOI, (**c**) BOC/BOI-1, (**d**) BOC/BOI-2, (**e**) BOC/BOI-3, and (**f**) BOC/BOI-4 photocatalysts.

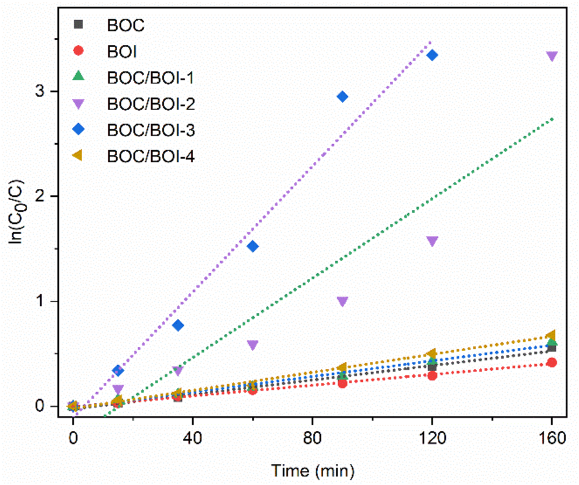

Figure S7. Pseudo first order reaction kinetic plots of BOC, BOI, BOC/BOI-1, BOC/BOI-2, BOC/BOI-3, and BOC/BOI-4 photocatalyst for RhB degradation.

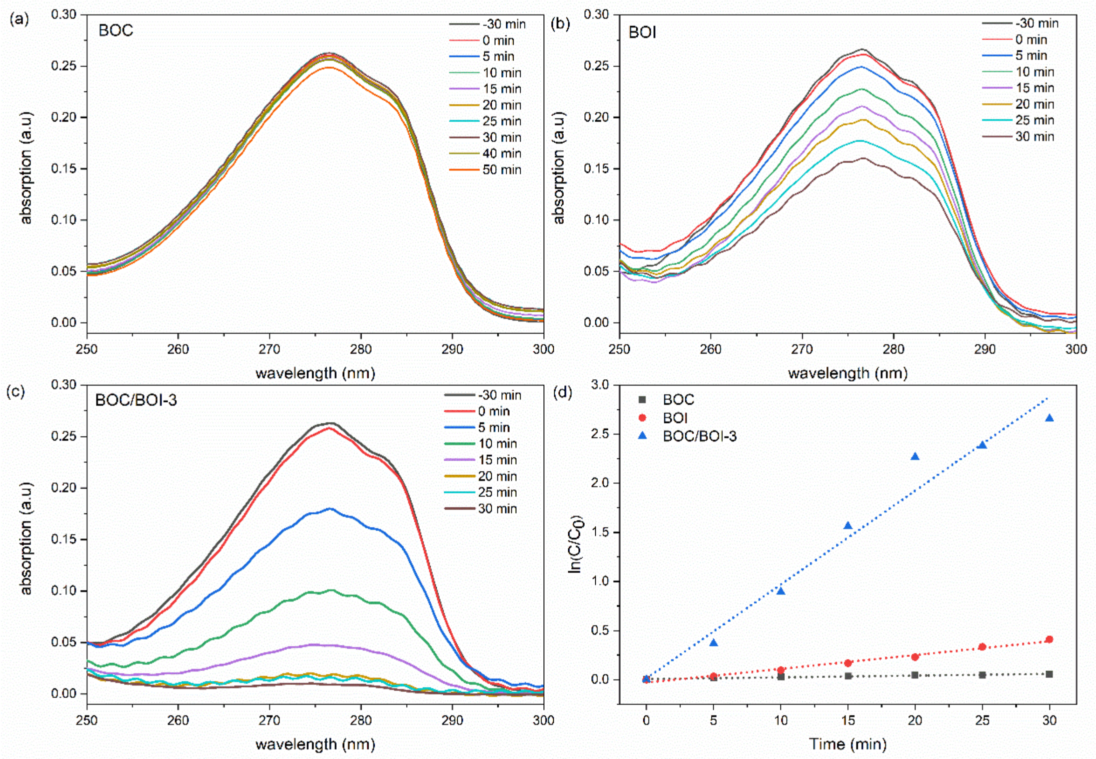

Figure S8. Time-resolved absorption spectra of RhB degradation with (**a**) BOC, (**b**) BOI,(**c**) BOC-BOI-3, and (**d**) Pseudo first order reaction kinetic plots of BOI, BOC, and BOC/BOI-3.

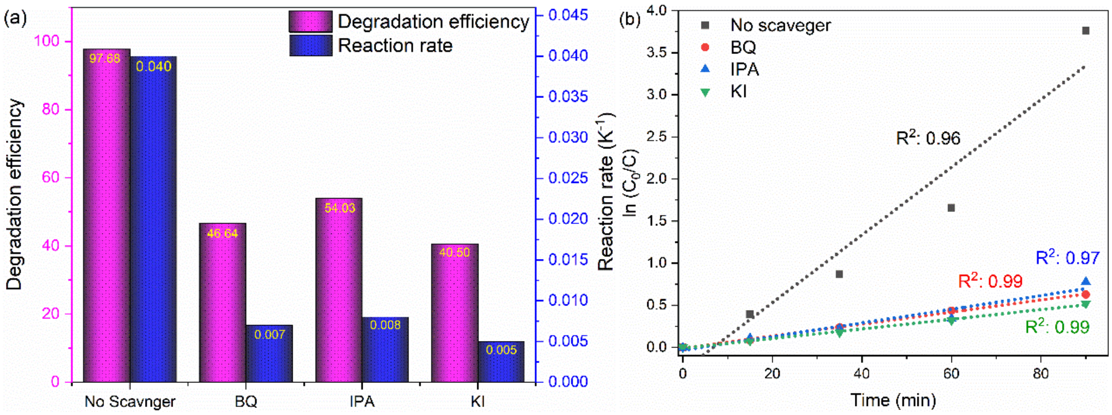

Figure S9. (**a**) Degradation efficiency with reaction rate constant and (**b**) 1st order reaction kinetics plot of RhB with and without scavengers in the presence of BOC/BOI-3.

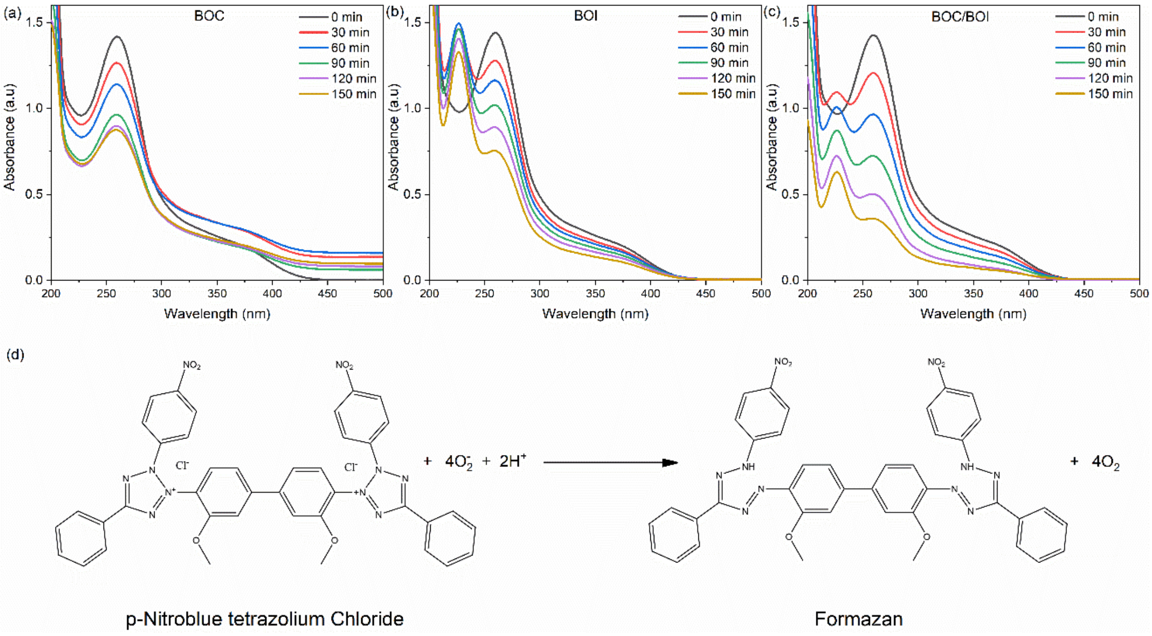

Figure S10. NBT degradation in the presence of (**a**) BOC, (**b**) BOI, and (**c**) BOC/BOI-3; (**d**) Chemical reaction for conversion of p-Nitroblue tetrazolium Chloride (NBT) to Formazan in the presence of super oxide radicals ($\bullet O_2^-$).

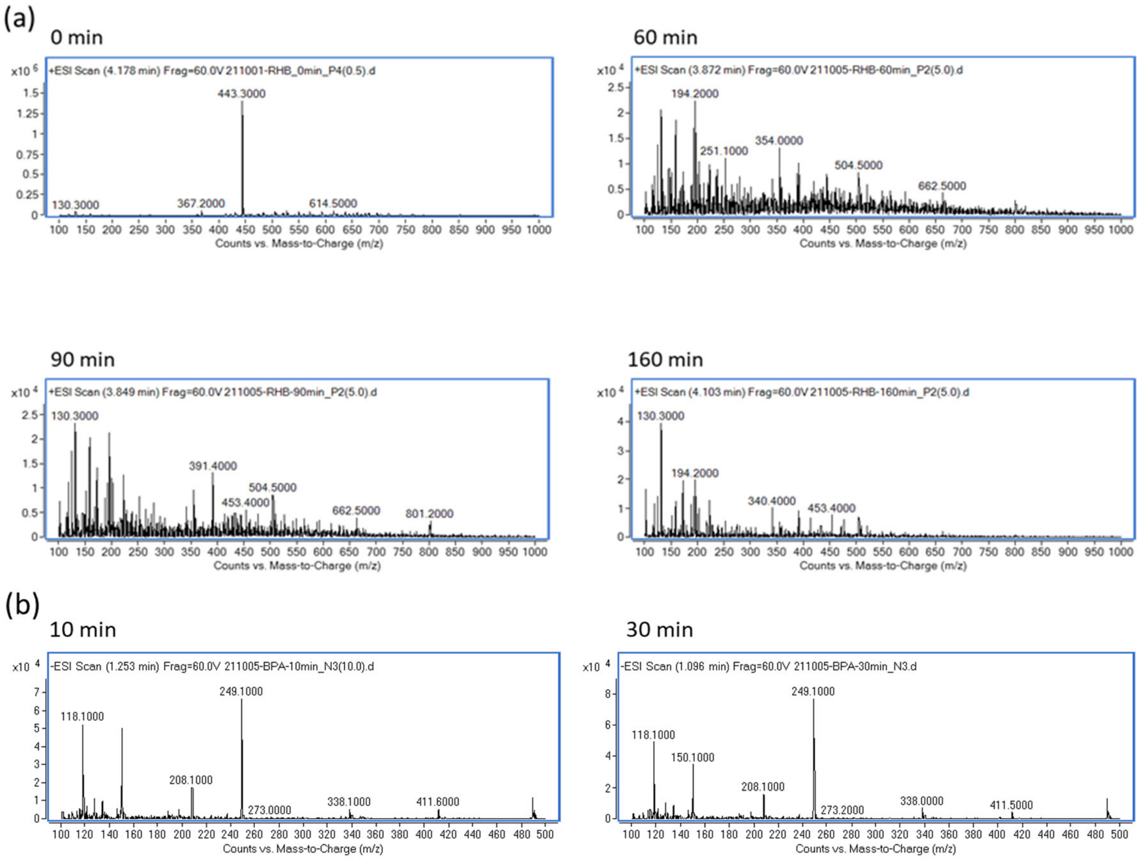

Figure S11. LC-MS of (**a**) RhB and (**b**) BPA after various photocatlytic reaction time.

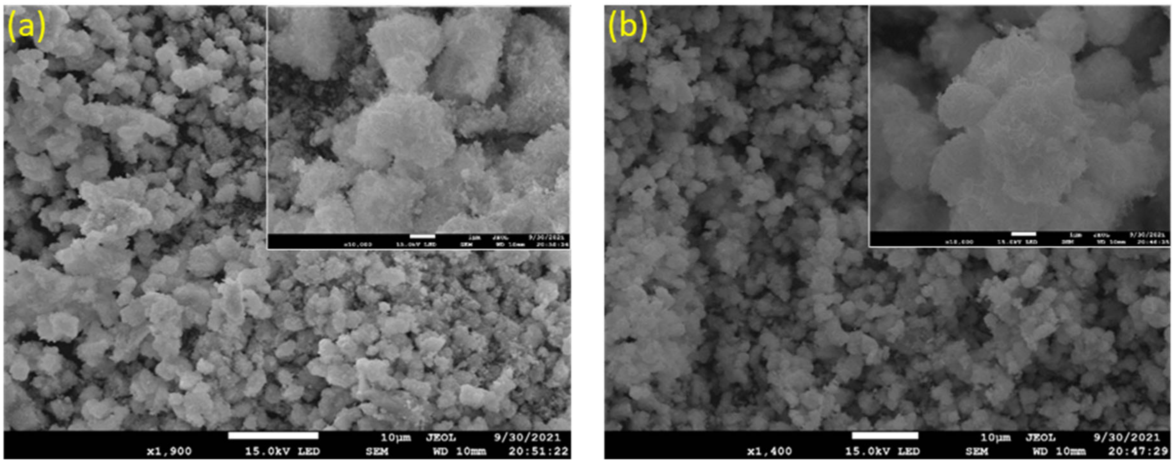

Figure S12. FESEM images BOC/BOI-3 heterostructures after (**a**) RhB and (**b**) BPA degradation.

Table S1. Obtained parameters from the TRPL curves.

Sample	A1	τ1	A2	τ1	<τ>
BOC *	5.64×10^{12}	0.53741	35.9181	7.42557	0.53741
BOI	2.09×10^{12}	0.5551	31.54899	7.91884	0.55511
BOC/BOI-3	3.26×10^{12}	0.92468	25.33757	8.80043	0.92474

Table S2. Comparison of photocatalytic degradation of RhB with recently reported literature.

Photocatalyst	Lamp (s)	Amount of catalyst (mg)	Solution volume (mL)	Solution concentration (mg.L^{-1})	Degradation time (min)	Rate constant K (min^{-1})	Removal rate (%)	Ref.
Bi_3O_4Cl/ Bi_5O_7I	300 W, Xe lamp (<420 nm)	10	40	17	90	0.040	97.68	This work
Bi_3O_4Cl/g-C_3N_4	350 W, Xe lamp (<420 nm)	100	100	10	120	0.019	91.5	[9]
Bi_3O_4Cl/$SrFe_{12}O_{19}$	300 W, Xe lamp	100	100	10	80	0.0387	99.15	[10]
AgCl/Bi_3O_4Cl	350 W, Xe lamp (<420 nm)	200	100	--	70	0.048	99.5	[11]
AgI/Bi_5O_7I	350 W, Xe lamp (<420 nm)	100	100	10	120	0.046	99	[12]
g-C_3N_4/Bi_5O_7I	300 W, Xe lamp (<420 nm)	40	40	10	20	0.197	98.2	[13]
g-C_3N_4/Bi_5O_7I	500 W, Xe lamp (<420 nm)	50	50	4.8	120	0.0186	90	[14]
Pal/Bi_5O_7I/$Bi_4O_5I_2$	300 W, Xe lamp (<420 nm)	50	100	10	40	0.08	99.9	[15]
Ag/AgBr/Bi_5O_7I	500 W, Xe lamp (<420 nm)	50	50	5	60	0.0557	95	[16]
$Bi_5O_7B_{0.5}I_{0.5}$	300 W, Xe lamp	50	100	20	120	--	68	[17]
BiOI/$Mn_xZn_{1-x}Fe_2O_4$	250 W, Xe lamp	100	100	10	240	--	59	[18]
$Bi_4O_5I_2$/Bi_5O_7I	500 W, Xe lamp (<420 nm)	50	50	9.6	240	--	79	[19]
Bi_3O_4Cl/g-C_3N_4	250 W, Xe lamp (<420 nm)	50	100	10	90	--	98.3	[20]

Table S3. Comparison of photocatalytic degradation of BPA with recently reported literature.

Photocatalyst	Lamp (s)	Amount of catalyst (mg)	Solution volume (mL)	Solution concentration (mg.L^{-1})	Degradation time (min)	Rate constant K (min^{-1})	Removal rate (%)	Ref.
Bi_3O_4Cl/ Bi_5O_7I	300 W Xe lamp (<420 nm)	40	40	15	30	0.094	92.8	This work
$Bi_4O_5Br_2/MnO_2$	300 W Xe lamp (<420 nm)	40	80	20	180	0.011	87	[21]
Er^{3+}/Fe^{3+} co-doped Bi_5O_7I	400 W Xe lamp (<420 nm)	50	50	15	18	0.2215	100	[22]
Bi_5O_7I/UiO-67	800 W, Xe lamp (<290 nm)	28	40	50	60	0.05513	99	[23]
$Bi_2O_2Co_3/Bi_5O_7I$	300 W Xe lamp (<400 nm)	50	100	10	60	0.16635	100	[24]
$Bi_{12}O_{17}Cl_2$/MIL-100(Fe)	300 W	25	200	10 mg.L^{-1}, ph:5.2 + 0.2 mM Na_2SO_4	60	0.14713	100	[25]
Bi_2S_3/g-C_3N_4	500 W Xe lamp (<320 nm)	30	50	15	240	0.2691	71	[26]
C/TiO_2	300 W (<420	20	100	20	180	0.01887	98	[27]
ZnO/BiOI	100 W Xe lamp (<420 nm)	25	50	10	120	0.0124	70	[28]
Bi_2MoO_6/MIL-88B(Fe)	350 W Xe lamp (<420 nm)		50	20	120	--	54	[29]
2D CN-$Br_{0.12}$/2%RhO_x	250 W Xe lamp (<420 nm)	10	15	10	9	--	100	[30]
Ag@$AgVO_3$/Bi-OCl	Day light	50	100	--	240	0.0265	52.8	[31]
CQDs/Bi_5O_7I	300 W Xe lamp (<400 nm)	50	100	10	120	--	94.2	[32]
$Bi_5O_7B_{0.5}I_{0.5}$	300 W	50	100	10	300	--	35	[17]
MoS_2/Bi_5O_7I	300 W Xe lamp (<400 nm)	50	100	10	60	--	99	[33]
$Bi_4O_5I_2/Bi_5O_7I$	500 W, Xe lamp (<400 nm)	50	50	10	240	--	94	[19]

References

1. Mian, F.; Bottaro, G.; Rancan, M.; Pezzato, L.; Gombac, V.; Fornasiero, P.; Armelao, L. $Bi_{12}O_{17}C_{12}/(BiO)_2CO_3$ Nanocomposite Materials for Pollutant Adsorption and Degradation: Modulation of the Functional Properties by Composition Tailoring. *ACS Omega* **2017**, *2*, 6298–6308, doi:10.1021/acsomega.7b01125.
2. Han, L.; Guo, Y.; Lin, Z.; Huang, H. 0D to 3D Controllable Nanostructures of BiOBr via a Facile and Fast Room-Temperature Strategy. *Colloids Surf. A: Physicochem. Eng. Asp.* **2020**, *603*, 125233, doi:10.1016/j.colsurfa.2020.125233.
3. Cui, B.; Cui, H.; Li, Z.; Dong, H.; Li, X.; Zhao, L.; Wang, J. Novel $Bi_3O_5I_2$ Hollow Microsphere and Its Enhanced Photocatalytic Activity. *Catal.* **2019**, *9*, 709, doi:10.3390/catal9090709.
4. Yu, C.; Fan, C.; Yu, J.C.; Zhou, W.; Yang, K. Preparation of Bismuth Oxyiodides and Oxides and Their Photooxidation Characteristic under Visible/UV Light Irradiation. *Mater. Res. Bull.* **2011**, *46*, 140–146, doi:10.1016/j.materresbull.2010.08.013.
5. Liao, C.; Ma, Z.; Chen, X.; He, X.; Qiu, J. Controlled Synthesis of Bismuth Oxyiodide toward Optimization of Photocatalytic Performance. *Appl. Surf. Sci.* **2016**, *387*, 1247–1256, doi:10.1016/j.apsusc.2016.06.140.
6. Sun, X.; Wu, J.; Li, Q.; Liu, Q.; Qi, Y.; You, L.; Ji, Z.; He, P.; Sheng, P.; Ren, J.; et al. Fabrication of $BiOIO_3$ with Induced Oxygen Vacancies for Efficient Separation of the Electron-Hole Pairs. *Appl. Catal. B: Environ.* **2017**, *218*, 80–90, doi:10.1016/j.apcatb.2017.06.041.
7. Mera, A.C.; Rodríguez, C.A.; Meléndrez, M.F.; Valdés, H. Synthesis and Characterization of BiOI Microspheres under Standardized Conditions. *J Mater Sci* **2017**, *52*, 944–954, doi:10.1007/s10853-016-0390-x.
8. Long, M.; Hu, P.; Wu, H.; Chen, Y.; Tan, B.; Cai, W. Understanding the Composition and Electronic Structure Dependent Photocatalytic Performance of Bismuth Oxyiodides. *J. Mater. Chem. A* **2015**, *3*, 5592–5598, doi:10.1039/C4TA06134A.
9. Ma, Y.; Chen, Y.; Feng, Z.; Zeng, L.; Chen, Q.; Jin, R.; Lu, Y.; Huang, Y.; Wu, Y.; He, Y. Preparation, Characterization of $Bi_3O_4Cl/g\text{-}C_3N_4$ Composite and Its Photocatalytic Activity in Dye Degradation. *J. Water Process Eng.* **2017**, *18*, 65–72, doi:10.1016/j.jwpe.2017.06.002.
10. Wang, H.; Xu, L.; Liu, C.; Jiang, Z.; Feng, Q.; Wu, T.; Wang, R. A Novel Magnetic Photocatalyst $Bi_3O_4Cl/SrFe_{12}O_{19}$: Fabrication, Characterization and Its Photocatalytic Activity. *Ceram. Int.* **2020**, *46*, 460–467, doi:10.1016/j.ceramint.2019.08.283.
11. Huang, Y.; He, Y.; Cui, M.; Nong, Q.; Yu, J.; Wu, F.; Meng, X. Synthesis of $AgCl/Bi_3O_4Cl$ Composite and Its Photocatalytic Activity in RhB Degradation under Visible Light. *Catal. Commun.* **2016**, *76*, 19–22, doi:10.1016/j.catcom.2015.12.018.
12. Cui, M.; Yu, J.; Lin, H.; Wu, Y.; Zhao, L.; He, Y. In-Situ Preparation of Z-Scheme AgI/Bi_5O_7I Hybrid and Its Excellent Photocatalytic Activity. *Appl. Surf. Sci.* **2016**, *387*, 912–920, doi:10.1016/j.apsusc.2016.07.024.
13. Geng, X.; Chen, S.; Lv, X.; Jiang, W.; Wang, T. Synthesis of $G\text{-}C_3N_4/Bi_5O_7I$ Microspheres with Enhanced Photocatalytic Activity under Visible Light. *Appl. Surf. Sci.* **2018**, *462*, 18–28, doi:10.1016/j.apsusc.2018.08.080.
14. Liu, C.; Huang, H.; Du, X.; Zhang, T.; Tian, N.; Guo, Y.; Zhang, Y. In Situ Co-Crystallization for Fabrication of $g\text{-}C_3N_4/Bi_5O_7I$ Heterojunction for Enhanced Visible-Light Photocatalysis. *J. Phys. Chem. C* **2015**, *119*, 17156–17165, doi:10.1021/acs.jpcc.5b03707.
15. Luo, Y.; Zhang, M.; Yin, H.; Yao, J.; Chen, S.; Liu, X. One Pot Controllable Synthesis of Palygorskite/Bismuth Oxyiodide Hierarchical Microspheres for Improved Visible-Light Photocatalytic Performance. *Colloids Surf. A: Physicochem. Eng. Asp.* **2019**, *578*, 123573, doi:10.1016/j.colsurfa.2019.06.038.
16. Zhao, Z.; Wang, M.; Yang, T.; Fang, M.; Zhang, L.; Zhu, H.; Tang, C.; Huang, Z. In Situ Co-Precipitation for the Synthesis of an $Ag/AgBr/Bi_5O_7I$ Heterojunction for Enhanced Visible-Light Photocatalysis. *J. Mol. Catal. A: Chem.* **2016**, *424*, 8–16, doi:10.1016/j.molcata.2016.08.004.
17. Bai, Y.; Shi, X.; Wang, P.; Wang, L.; Xie, H.; Li, Z.; Qu, L.; Ye, L. Synthesis of One-Dimensional $Bi_5O_7Br_{0.5}I_{0.5}$ Solid Solution for Effective Real Oilfield Wastewater Treatment via Exciton Photocatalytic Process. *J. Taiwan Inst. Chem. Eng.* **2018**, *91*, 358–368, doi:10.1016/j.jtice.2018.05.045.

18. Feng, S.; Du, H.; Xie, T.; Xu, L.; Wang, Y. Preparation and Photocatalytic Activity of BiOI/$Mn_xZn_{1-x}Fe_2O_4$ Magnetic Photocatalyst. *Ceram. Int.* **2019**, *45*, 10468–10474, doi:10.1016/j.ceramint.2019.02.108.
19. Huang, H.; Xiao, K.; Zhang, T.; Dong, F.; Zhang, Y. Rational Design on 3D Hierarchical Bismuth Oxyiodides via in Situ Self-Template Phase Transformation and Phase-Junction Construction for Optimizing Photocatalysis against Diverse Contaminants. *Appl. Catal. B: Environ.* **2017**, *203*, 879–888, doi:10.1016/j.apcatb.2016.10.082.
20. Che, H.; Che, G.; Dong, H.; Hu, W.; Hu, H.; Liu, C.; Li, C. Fabrication of Z-Scheme Bi_3O_4Cl/g-C_3N_4 2D/2D Heterojunctions with Enhanced Interfacial Charge Separation and Photocatalytic Degradation Various Organic Pollutants Activity. *Appl. Surf. Sci.* **2018**, *455*, 705–716, doi:10.1016/j.apsusc.2018.06.038.
21. Chang, F.; Yan, W.; Wang, X.; Peng, S.; Li, S.; Hu, X. Strengthened Photocatalytic Removal of Bisphenol a by Robust 3D Hierarchical N-p Heterojunctions $Bi_4O_5Br_2$-MnO_2 via Boosting Oxidative Radicals Generation. *Chem. Eng. J.* **2022**, *428*, 131223, doi:10.1016/j.cej.2021.131223.
22. Liu, Y.; Zhu, G.; Gao, J.; Zhu, R.; Hojamberdiev, M.; Wang, C.; Wei, X.; Liu, P. A Novel Synergy of Er^{3+}/Fe^{3+} Co-Doped Porous Bi_5O_7I Microspheres with Enhanced Photocatalytic Activity under Visible-Light Irradiation. *Appl. Catal. B: Environ.* **2017**, *205*, 421–432, doi:10.1016/j.apcatb.2016.12.061.
23. Cao, C.-S.; Wang, J.; Yu, X.; Zhang, Y.; Zhu, L. Photodegradation of Seven Bisphenol Analogues by Bi_5O_7I/UiO-67 Heterojunction: Relationship between the Chemical Structures and Removal Efficiency. *Appl. Catal. B: Environ.* **2020**, *277*, 119222, doi:10.1016/j.apcatb.2020.119222.
24. Guo, Y.; Nan, J.; Xu, Y.; Cui, F.; Shi, W.; Zhu, Y. Thermodynamic and Dynamic Dual Regulation $Bi_2O_2CO_3$/Bi_5O_7I Enabling High-Flux Photogenerated Charge Migration for Enhanced Visible-Light-Driven Photocatalysis. *J. Mater. Chem. A* **2020**, *8*, 10252–10259, doi:10.1039/D0TA02588G.
25. Zhao, C.; Wang, J.; Chen, X.; Wang, Z.; Ji, H.; Chen, L.; Liu, W.; Wang, C.-C. Bifunctional Bi12O17Cl2/MIL-100(Fe) Composites toward Photocatalytic Cr(VI) Sequestration and Activation of Persulfate for Bisphenol A Degradation. *Science of The Total Environment* **2021**, *752*, 141901, doi:10.1016/j.scitotenv.2020.141901.
26. Gu, J.; Yu, Y.; Chen, S.; Shi, W.; Wang, Y.; Liao, Y.; Chen, H.; Jiang, F. Heterojunction Photocatalyst of Cavity Shaped Bi_2S_3/g-C_3N_4 for Bisphenol a Degradation: Regulation of Internal Electric Field via Assistance of Interfacial Functional Groups. *Chem. Eng. J.* **2021**, *424*, 130539, doi:10.1016/j.cej.2021.130539.
27. He, X.; Wu, M.; Ao, Z.; Lai, B.; Zhou, Y.; An, T.; Wang, S. Metal–Organic Frameworks Derived C/TiO_2 for Visible Light Photocatalysis: Simple Synthesis and Contribution of Carbon Species. *J. Hazard. Mater.* **2021**, *403*, 124048, doi:10.1016/j.jhazmat.2020.124048.
28. Feng, H.; Liang, L.; Wu, W.; Huang, Z.; Liu, Y. Architecting Epitaxial-Lattice-Mismatch-Free (LMF) Zinc Oxide/Bismuth Oxyiodide Nano-Heterostructures for Efficient Photocatalysis. *J. Mater. Chem. C* **2020**, *8*, 11263–11273, doi:10.1039/D0TC02607G.
29. Zhao, K.; Zhang, Z.; Feng, Y.; Lin, S.; Li, H.; Gao, X. Surface Oxygen Vacancy Modified Bi_2MoO_6/MIL-88B(Fe) Heterostructure with Enhanced Spatial Charge Separation at the Bulk & Interface. *Appl. Catal. B: Environ.* **2020**, *268*, 118740, doi:10.1016/j.apcatb.2020.118740.
30. Fei, H.; Shao, J.; Li, H.; Li, N.; Chen, D.; Xu, Q.; He, J.; Lu, J. Construction of Ultra-Thin 2D CN-$Br_{0.12}$/2%RhO_x Photo-Catalyst with Rapid Electron and Hole Separation for Efficient Bisphenol A Degradation. *Appl. Catal. B: Environ.* **2021**, *299*, 120623, doi:10.1016/j.apcatb.2021.120623.
31. Kuila, A.; Saravanan, P.; Bahnemann, D.; Wang, C. Novel Ag Decorated, BiOCl Surface Doped $AgVO_3$ Nanobelt Ternary Composite with Z-Scheme Homojunction-Heterojunction Interface for High Prolific Photo Switching, Quantum Efficiency and Hole Mediated Photocatalysis. *Appl. Catal. B: Environ.* **2021**, *293*, 120224, doi:10.1016/j.apcatb.2021.120224.

32. Chen, R.; Chen, Z.; Ji, M.; Chen, H.; Liu, Y.; Xia, J.; Li, H. Enhanced Reactive Oxygen Species Activation for Building Carbon Quantum Dots Modified Bi_5O_7I Nanorod Composites and Optimized Visible-Light-Response Photocatalytic Performance. *J. Colloid Interface Sci.* **2018**, *532*, 727–737, doi:10.1016/j.jcis.2018.07.027.
33. Yin, S.; Chen, R.; Ji, M.; Jiang, Q.; Li, K.; Chen, Z.; Xia, J.; Li, H. Construction of Ultrathin MoS_2/Bi_5O_7I Composites: Effective Charge Separation and Increased Photocatalytic Activity. *J. Colloid Interface Sci.* **2020**, *560*, 475–484, doi:10.1016/j.jcis.2019.10.081.
